# Retraction Note: Alginate oligosaccharide attenuates α2,6- sialylation modification to inhibit prostate cancer cell growth via the Hippo/YAP pathway

**DOI:** 10.1038/s41419-021-04425-w

**Published:** 2021-12-09

**Authors:** Yang Han, Lin Zhang, Xiao Yu, Shidan Wang, Chunyan Xu, Heng Yin, Shujing Wang

**Affiliations:** 1 Department of Biochemistry and Molecular Biology, College of Basic Medical Sciences, Institute of Glycobiology, Dalian Medical University, Dalian, Liaoning China; 2 Department of Pathology, College of Basic Medical Sciences, Dalian Medical University, Dalian, Liaoning China; 3 Liaoning Provincial Key Laboratory of Carbohydrates, Dalian Institute of Chemical Physics, Chinese Academy of Science, Dalian, China

**Keywords:** Cancer prevention, Glycobiology, Glycobiology

Retraction Note to: *Cell Death & Disease* 10.1038/s41419-019-1560-y, published online 10 May 2019

The editors-in-chief have retracted this article because of anomalies in two of the figures. Specifically:

In figure 2C the DU145/ 500 µg/ml AOS panel appears to be the same as the PC3/ 500 µg/ml AOS panel of figure 2D.

In figure 5A the MST1/ DU145 panel appears to be the same as the PC3/LATS1 panel.

The editors-in-chief therefore no longer have confidence in the data presented in this study.

Shujing Wang, Yang Han, Lin Zhang and Chunyan Xu disagree with this retraction. Xiao Yu, Shidan Wang and Heng Yin have not responded to any correspondence from the Editors or the Publisher about this retraction.

